# Supplementary material for: MetaRibo-Seq measures translation in microbiomes
Source: Nat Commun. 2020 Jun 29;11:3268. doi: 10.1038/s41467-020-17081-z (PMC7324362; doi:10.1038/s41467-020-17081-z)
Supplement: Supplementary file 10 — Supplementary Data 7 [file 41467_2020_17081_MOESM10_ESM.zip › File2/Confidence_High_Taxonomy/1307_out.krona.html]

Javascript must be enabled to view this page.

members
magnitude
magnitudeUnassigned
count
unassigned
taxon
rank

1307\_out

53

superkingdom
2
53

phylum
976
53

class
200643
53

53
171549
order

53
815
family

53
816
genus

species
371601

SRS011405\_contig\_number\_14724SRS013687\_contig\_number\_30408SRS013951\_contig\_number\_contig-100\_1411.232405SRS016132\_contig\_number\_contig-100\_2717.38691SRS016495\_contig\_number\_14871SRS016753\_contig\_number\_4669SRS016989\_contig\_number\_contig-100\_2432.96244SRS017247\_contig\_number\_contig-100\_2747.64051SRS017521\_contig\_number\_5512SRS017859\_contig\_number\_contig-100\_2135.51246SRS018049\_contig\_number\_2556SRS018351\_contig\_number\_17988SRS019161\_contig\_number\_contig-100\_523.150311SRS019286\_contig\_number\_5358SRS019968\_contig\_number\_contig-100\_1881.110496SRS020622\_contig\_number\_contig-100\_2409.50964SRS023914\_contig\_number\_24209SRS024009\_contig\_number\_11280SRS024075\_contig\_number\_12824SRS024388\_contig\_number\_10732SRS045713\_contig\_number\_21356SRS046369\_contig\_number\_10875SRS047433\_contig\_number\_contig-100\_718.141754SRS048981\_contig\_number\_4318SRS049402\_contig\_number\_2610SRS049896\_contig\_number\_contig-100\_2137.195700SRS050026\_contig\_number\_10143SRS052027\_contig\_number\_contig-100\_3253.108476SRS052078\_contig\_number\_contig-100\_2519.39266SRS053214\_contig\_number\_12443SRS056273\_contig\_number\_13373SRS063489\_contig\_number\_contig-100\_3547.39201SRS063813\_contig\_number\_contig-100\_1857.59088SRS074670\_contig\_number\_5550SRS075078\_contig\_number\_1321SRS077454\_contig\_number\_15385SRS077730\_contig\_number\_10620SRS097889\_contig\_number\_39732SRS101268\_contig\_number\_contig-100\_562.14481SRS1041112\_contig\_number\_contig-100\_2774.44446SRS1041116\_contig\_number\_3647SRS1041130\_contig\_number\_contig-100\_3494.44131SRS104636\_contig\_number\_32199SRS1055049\_contig\_number\_17327SRS1055069\_contig\_number\_14316SRS140492\_contig\_number\_contig-100\_2797.193619SRS142599\_contig\_number\_27463SRS143722\_contig\_number\_7877SRS143780\_contig\_number\_2990SRS144362\_contig\_number\_40680SRS147139\_contig\_number\_contig-100\_3365.224272SRS149879\_contig\_number\_contig-100\_762.159933
52

46506
species

SRS1041132\_contig\_number\_6203
1
